# Viscoelastometry to Manage Bleeding in Liver Disease

**DOI:** 10.7759/cureus.41401

**Published:** 2023-07-05

**Authors:** Samantha Wilson, Joanne Joseph, Mark Danta, David J Rabbolini

**Affiliations:** 1 Gastroenterology and Hepatology, School of Clinical Medicine, St. Vincent’s Healthcare Campus, Faculty of Medicine, University of New South Wales, Sydney, AUS; 2 Hematology, School of Clinical Medicine, St. Vincent’s Healthcare Campus, Faculty of Medicine, University of New South Wales, Sydney, AUS; 3 Hematology, St. Vincent’s Centre for Applied Medical Research, St Vincent’s Hospital, Sydney, AUS; 4 Gastroenterology and Hepatology, St. Vincent’s Hospital, Sydney, AUS; 5 Faculty of Medicine and Health, University of Sydney, Sydney, AUS; 6 Haematology, Oxford University Hospitals NHS Foundation Trust, Oxford, GBR

**Keywords:** thrombosis, bleeding, cirrhosis, liver disease, viscoelastometry

## Abstract

A state of “re-balanced haemostasis” describes complex coagulation changes that arise in patients with liver disease. Changes include alterations in procoagulant and anticoagulant proteins, platelets and von Willebrand factor, as well as the fibrinolytic system. Various circumstances including infection, trauma, or surgery may disrupt this balance and predispose an individual to bleeding or thrombosis. The prothrombin time, international normalised ratio, and activated partial thromboplastin time are conventional coagulation screening tests that are routinely employed by clinicians to investigate unexplained bleeding, monitor anticoagulation, and inform preoperative assessments of bleeding risk. These standard coagulation tests assess quantitative defects in procoagulant clotting factors and are insensitive to levels of natural anticoagulants, which together with procoagulant factors, are often perturbed in liver disease. Therefore, the prolongation of clotting times measured by these tests often does not reflect the multifaceted alterations of haemostasis in these patients. Viscoelastic testing (VET) provides a more encompassing assessment of clotting function by recording real-time viscoelastic changes in whole blood and includes parameters that provide information on coagulation factor function, platelet contribution to clot formation, as well as fibrinolysis. To date, VET has been employed to predict and inform transfusion support in obstetric, trauma, and cardiac surgical fields, and its use in patients undergoing liver transplantation is well established. The ability of VET to accurately predict bleeding risk and precisely guide transfusion algorithms for patients with liver disease undergoing other invasive procedures or experiencing bleeding complications has been the topic of research over the last decade. This review is a critical summary of this data and provides a detailed snapshot of the position of VET as a clinical tool in patients with liver disease.

## Introduction and background

Liver disease may affect the liver’s critical role in synthesising proteins that are important for haemostasis. These proteins include coagulation factors (e.g., factors II, V, VII, IX, X, XI, XIII, and fibrinogen), natural anticoagulants (Protein C, Protein S, antithrombin (AT), and tissue factor pathway inhibitor (TFPI)), cytokines (thrombopoietin), profibrinolytic proteins (plasminogen and tissue plasminogen activator (tPA)), and antifibrinolytic proteins (plasminogen activator inhibitor-1 (PAI-1), alpha 2-antiplasmin (A2AP), and thrombin activatable fibrinolysis inhibitor (TAFI)). Moreover, liver parenchymal changes occurring in cirrhosis may cause altered portal venous drainage into the liver. Resultant portal venous hypertension may cause splenomegaly and ultimately thrombocytopenia from increased platelet sequestration in the spleen. Consequently, haemostatic derangements in liver disease are often multifactorial encompassing changes in both primary and secondary haemostatic elements, compounded by alterations in the portal circulation. The assessment of haemostasis in liver disease has traditionally used standard coagulation tests (SCTs) such as the prothrombin time (PT), activated partial thromboplastin time (aPTT), fibrinogen, as well as platelet count. In cases where these parameters are deranged, transfusions of fresh frozen plasma (FFP), cryoprecipitate, and/or platelets have been employed to reduce perceived bleeding risks [1]. Unfortunately, abnormalities of SCTs in patients with altered liver function are a poor reflection of global haemostasis and bleeding risk [2]. Point-of-care (POC) viscoelastic testing (VET) using platforms such as rotational thromboelastometry (ROTEM), thromboelastography (TEG), ClotPro, and Quantra provide global measures of coagulation and have been increasingly used to attempt to better define patients with liver disease who are at an increased risk of bleeding and/or clotting, as well as to rationalise transfusion of blood products in patients actively bleeding and during invasive procedures [3]. This narrative review will discuss the effects of deranged liver function on haemostasis, the principles of VET, and the utility of VET in predicting and managing bleeding in liver disease.

## Review

Haemostasis in liver disease

**Figure 1:**
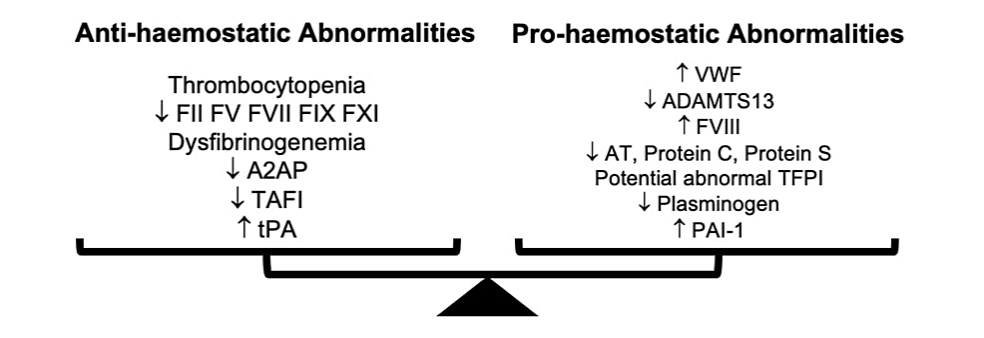
Opposing pro- and anti-haemostatic forces balance each other in liver disease. FII, FV, FVII, FVIII, FIX, FXI = coagulation factors; ADAMTS13 = a disintegrin and metalloproteinase with a thrombospondin type 1 motif, member 13; A2AP = alpha 2-antiplasmin; TAFI  = thrombin activatable fibrinolysis inhibitor; tPA = tissue plasminogen activator; VWF  = von Willebrand factor; AT = antithrombin; TFPI = tissue factor pathway inhibitor; PAI-1 = plasminogen activator inhibitor-1

Liver disease may disrupt pro- and anti-haemostatic factors that involve components of primary haemostasis (platelets, fibrinogen and von Willebrand factor (VWF)), secondary haemostasis (coagulation proteins), and fibrinolysis, resulting in a state of abnormal but “re-balanced haemostasis” (Figure 1). Thrombocytopenia occurs because of low thrombopoietin levels and platelet sequestration from portal hypertension-related hypersplenism. Thrombocytopenia, in turn, may be partially compensated by increased VWF, which is commonly raised in liver disease. The cause of increased VWF is thought to be related to endothelial perturbation occurring in part from endotoxaemia, a consequence of bacterial translocation/systemic inflammation and reduced clearance from a damaged liver [4-6]. Fibrinogen levels may be reduced, normal, or increased in patients with liver disease. A large proportion of patients may also develop qualitative abnormalities of fibrinogen characterized by dysfunctional fibrin monomer polymerization caused by the synthesis of altered fibrinogen containing increased sialic acid residues from injured hepatocytes [7,8].

Reductions of coagulation factors, as well as their natural inhibitors, are well established in liver disease, but overall, thrombin generation is thought to be preserved [1,9]. Production and clearance of pro- and anti-fibrinolytic proteins may also be altered in liver disease causing disturbances in fibrinolysis. A hyperfibrinolytic state, observed in 30-50% of patients, may be caused by an underproduction of fibrinolytic inhibitors such as A2AP and TAFI with subsequent increased plasmin generation [10]. Hyperfibrinolysis may be exacerbated by insults such as sepsis as well as the reabsorption of ascitic fluid with fibrinolytic activity into the systemic circulation [11]. Conversely, a hypofibrinolytic state may be induced by substantially increased PAI-1 in acute liver failure [10]. Overall, a fragile balance between pro-thrombotic and pro-haemorrhagic states is generated [12]. Disruption of this balance may cause bleeding, commonly from oesophageal and/or gastric varices, or thrombosis [13]. Common sites of thrombosis in patients with cirrhosis include the portal and mesenteric veins, as well as deep vein thrombosis and pulmonary embolism [13].

Standard coagulation tests in liver cirrhosis

SCTs, in general, comprise tests that provide information about coagulation (PT, aPTT, INR, and fibrinogen). Platelet count is also included in initial assessments. Qualitative measurements of platelet function or measurements of the fibrinolytic system and VWF are not routinely performed in initial haemostatic assessments. PT measures the time taken (in seconds) for blood to clot following the addition of activators of the extrinsic pathway (thromboplastin and calcium) to a citrated sample of plasma. PT prolongation may indicate deficiencies in factors I, II, V, VII, IX, and X. PT is insensitive to abnormalities of anti-coagulant proteins that are commonly perturbed in liver disease, including Protein C, Protein S, AT, and TFPI [2]. The international normalised ratio (INR) is a derived variable that corrects for interlaboratory variability of the PT caused by variations in analytical systems and reagents employed by different laboratories. It is calculated as a ratio of the patient’s PT to a control PT, multiplied by an international sensitivity index (ISI), which reflects the potency of the thromboplastin reagent. The INR is typically used to monitor individuals receiving anti-coagulation with vitamin K antagonists. The aPTT is commonly employed to evaluate intrinsic coagulation pathway components. This test measures the time taken for a citrated plasma sample to clot after the addition of a platelet substitute, (factor XII activator and calcium chloride). In patients with liver disease, neither the PT, INR, nor aPTT are predictive of bleeding complications [1,14-22]. In a prospective study by Massicotte et al., 200 patients undergoing liver transplantation were followed to determine whether preoperative coagulation defects influenced the requirement for red blood cell (RBC) transfusion [18]. The cohort was divided into a low INR (<1.5) group and a high INR (>1.5) group. In the absence of uncontrollable bleeding, coagulation abnormalities were not corrected before or during transplantation. The trigger for RBC transfusion was a haemoglobin of 60 g/L. Overall, 51.5% of patients had preoperative INR >1.5, 23% had an INR >2, and 6.5% had an INR >3. Like those with an INR <1.5, none of the patients in the high INR group required transfusion with FFP. Moreover, 81.5% of patients did not receive any RBC transfusion. The preoperative INR did not influence the transfusion rate (RBC units transfused, blood loss, percentage of cases without blood products, or the final haemoglobin value) [18].

Measurement of fibrinogen levels is used to estimate this substrate’s availability for clot formation. Decreased fibrinogen commonly occurs in cirrhosis due to reduced hepatic production and increased consumption, thus increasing the bleeding risk [10,23]. There is currently insufficient evidence to support fibrinogen supplementation in patients with liver disease, and current guidance among professional organisations lacks consistency. The American Gastroenterological Association (AGA) guidelines for coagulation in cirrhosis recommends a level above 120 mg/dL be targeted for optimal clot formation in active bleeding or before high-risk procedures [24]. The American Association for the Study of Liver Diseases (AASLD) 2020 practice guidance document suggests that correction of fibrinogen to >100 mg/dL using cryoprecipitate or fibrinogen concentrate before high-risk procedures could be considered in patients with cirrhosis [25]. In contrast, the recent clinical practice guideline released by the European Association for the Study of the Liver (EASL) discourages the correction of fibrinogen [26].

Thrombocytopenia may be associated with platelet dysfunction in liver diseases and together these factors may increase bleeding risk [27]. However, the platelet count at which increased bleeding is conferred and therefore maintained through transfusion is not clear. In a seminal study of 200 patients undergoing a laparoscopic liver biopsy, a platelet count below 30 × 10^9^/L was found to be a poor predictor of bleeding risk. The same result was found for PT, whole blood clot time, length of biopsy cylinder, and liver histopathology. In this study, the 10 patients with abnormal liver bleeding times (LBTs) (LBT >12 minutes) had no significant differences in platelet count compared to those whose LBTs fell within the normal range [28]. In another study, Vieira da Rocha et al. explored whether coagulation status could predict bleeding from post-banding ulcers, a complication affecting 5-10% of patients undergoing oesophageal variceal band ligation (EVL). Patients were divided into high (platelet count <50 × 10^9^/L and INR >1.5) and low (platelet count >50 × 10^9^/L and INR <1.5) bleeding risk groups. Post-EVL ulcer bleeding occurred in 7.3% (n = 11) of the cohort and was independent of coagulation test results [29]. The AGA’s guide on surgical risk assessment and perioperative management in cirrhosis and the EASL guideline on the prevention and management of bleeding and thrombosis in patients with cirrhosis suggest that platelet counts >50 × 10^9^/L are adequate for clotting in most cirrhotic patients. They add that prophylactic platelet transfusions to increase platelet count beyond that threshold are not likely to be beneficial to prevent bleeding complications [26,30]. This threshold is consistent with conclusions from an earlier large retrospective study of 5,987 patients that assessed patients undergoing percutaneous liver biopsies at the Mayo Clinic [31]. Here, a platelet count <50 × 10^9^/L was found to be a statistically significant risk factor for post-biopsy haemorrhage. The authors of that study concluded that, in the absence of other risk factors, such as more than two biopsy passes, a platelet count exceeding 50 × 10^9^/L was safe [31].

Laboratory tests used to measure platelet function are often multi-step and complex [32,33]. Many of the tests require staff with specific expertise to conduct them and analyse their results. In addition, the analytical precision and accuracy of most performed platelet function tests (the platelet function assay (PFA-100/200), light transmission aggregometry, whole blood aggregometry) are affected by thrombocytopenia rendering them unsuitable for patients with liver disease and thrombocytopenia [34,35]. Therefore, assessment of platelet function is not commonly performed in initial (or even subsequent) assessments of haemostasis in patients with liver disease. At present, it is unclear how laboratory results of platelet function in liver disease should be interpreted, and their predictive value for bleeding complications remains unclear [36,37].

To summarise, the limitations of SCTs to predict bleeding in patients with liver disease are broadly recognised and have prompted investigations into the ability of VET to better inform clinicians in the field.

Principles of viscoelastic tests

Viscoelastometry provides a measure of whole-blood haemostasis by recording viscoelastic changes in whole blood. HemoSonics’ Quantra viscoelastometry instruments use the sonic estimation of elasticity via resonance sonorheometry to generate information on clot formation, while all other platforms (ROTEM, TEG, ClotPro) measure changes in viscoelasticity induced by the blood clot’s physical impedance of a rotating pin or cup. Real-time traces of clot formation (including the rate of formation and maximal clot firmness) and clot breakdown are generated by each instrument enabling the user to directly visualise the haemostatic process (Table 1). Specific coagulation tests that isolate and quantify components of coagulation are generated by these viscoelastic platforms by exposing the recalcified whole blood sample to specific activators and inhibitors. Examples of tests employed by two instruments, ROTEM and ClotPro, in the setting of liver disease are the EXTEM (ROTEM) and EX-test (ClotPro) that test the extrinsic pathway, INTEM (ROTEM) and IN-test (ClotPro) that test the intrinsic pathway, FIBTEM (ROTEM) and FIB-test (ClotPro) that assess fibrinogen contribution, and APTEM (ROTEM) and AP-test (ClotPro) that evaluate fibrinolysis. NATEM (ROTEM) and NA-test (ClotPro) have also been used by some investigators. These assays measure coagulation in the sample following recalcification but without the addition of specific activators of coagulation.

**Table 1:** Parameter definitions for commonly used viscoelastic testing products.

TEG [38]		ROTEM [38] and ClotPro [39]	
Reaction (R) time	Time from start to 2 mm above baseline. Indicates the time for the clot to start forming	Clotting time (CT)	Time from start to reach 2 mm above baseline
Kinetics (K) time	Time from 2 mm to 20 mm above baseline	Clot formation time (CFT) (ROTEM only)	Time from 2 mm to 20 mm above baseline
Alpha angle	Slope between R and K points. Indicates the speed of fibrin accumulation	Alpha angle (ROTEM only)	Angle of the tangent at 2 mm amplitude, indicates the speed of fibrin accumulation
Maximum amplitude (MA)	Maximum strength of the clot, the highest vertical amplitude of the trace	Maximum clot firmness (MCF). Amplitude at 5 or 10 minutes (A5/A10)	Maximum strength of the clot, the highest vertical amplitude of the trace. Strength of the clot at the specified time
Percent rate of lysis (Ly30)	Percentage of amplitude reduction 30 minutes after the maximum amplitude	Lysis index 30 minutes after CT (LI30). Maximum lysis (ML)	Given as a percentage, the percent reduction in MCF 30 minutes after CT. Percentage of clot degraded in relation to MCF at any given point in time
Quantra [40]			
Clot time (CT)	Clotting activated by kaolin (activator of the intrinsic pathway)		
Clot stiffness (CS)	Stiffness of the blood clot after activation of the extrinsic pathway by thromboplastin and a heparin inhibitor (polybrene)		
Fibrinogen contribution to clot stiffness (FCS)	Measurement of functional fibrinogen’s contribution to overall clot stiffness after adding platelet inhibitor (abciximab)		
Platelet contribution to clot stiffness (PCS)	Clot stiffness minus fibrinogen contribution to clot stiffness		

There are currently five viscoelastic instruments that are commercially available and used clinically. The HemoSonics’ Quantra, ROTEM Sigma, and Haemonetics’ TEG instruments are cartridge-based providing simple and fast platforms for sample loading. The only requirement of the technician is the inversion and insertion of the blood collecting tube into the cartridge which is docked in the instrument. The single cartridge is divided into separate chambers containing reagents required for the specific coagulation tests. Unlike these cartridge-based platforms, the Haemonetics ClotPro and ROTEM Delta instruments comprise a stage that contains four or six separate wells, respectively, that house individual cups into which the blood sample is pipetted. The ROTEM Delta uses liquid reagents that require refrigeration and several pipetting steps before the addition of the blood sample. In contrast, ClotPro uses assay-specific pipette tips which contain all the reagents in dry form that are mixed with the blood when drawn up in the tip. This significantly reduces the complexity, and these tips are stable at room temperature for one month. While the pipetting steps of the ROTEM Delta and ClotPro add a level of complexity not shared by the aforementioned instruments, the ability to customise testing and repeat just one test is a significant benefit in terms of cost.

Viscoelastic tests to predict bleeding in liver disease

Coagulation tests that reliably predict bleeding risk could have the potential to improve patient outcomes and reduce blood product use and healthcare costs. Data are beginning to emerge on the ability of VET to predict bleeding risk in patients with liver disease. In a prospective cohort study by Seeßle et al. [41], investigators undertook the haemostatic assessment of patients with stable liver cirrhosis (non-acute-on-chronic liver failure) and patients with acute-on-chronic liver failure (ACLF) using SCTs and ROTEM. Subgroup analysis of patients with ACLF divided the cohort into those with bleeding (n = 9) during their hospital admission and those without bleeding events (n = 13) and correlated these outcomes to admission ROTEM and SCT measurements. In the bleeding group, significantly lower A10 was seen in all tests (NATEM median = 30.5 mm vs. 39.9 mm, p = 0.01; INTEM median = 33.0 mm vs. 38.0 mm, p < 0.0001; and EXTEM median = 32.0 vs. 39.0, p = 0.50), MCF was significantly lower in NATEM (median = 36.5 mm vs. 49.0 mm, p = 0.045) and INTEM (median = 65.0 mm vs. 45.0 mm, p = 0.045), and fibrinogen was significantly reduced (1.5 g/L vs. 1.9 g/L, p = 0.05) when compared with the non-bleeding group [41]. Overall, the authors concluded that ROTEM A10 and MCF represented suitable prognostic parameters in predicting bleeding events in patients with ACLF.

In the setting of liver transplantation, Dotsch et al. [42] showed that VET using ROTEM was able to accurately identify patients at risk of postoperative bleeding. This retrospective, single-centre, observational study of 243 patients undergoing liver transplantation compared the ability of SCTs and ROTEM VET to predict postoperative non-surgical bleeding. Blood samples were taken from all patients on admission to the intensive care unit (ICU) following surgery and were categorised as bleeders or non-bleeders subject to whether they received three or more RBC units (n = 3) or required reoperation for non-surgical bleeding (n = 23) within 48 hours of admission. Both SCT tests as well as ROTEM predicted bleeding (area under the curve (AUC) > 0.6). The best predictor of bleeding was with the EXTEM CT (AUC = 0.682). This test is equivalent to the PT. The INTEM CFT (AUC = 0.615), FIBTEM A10 (AUC = 0.615), and FIBTEM MCF (AUC = 0.611) also predicted postoperative bleeding.

El-Makarem et al. evaluated the ability of ROTEM to predict spontaneous oesophageal variceal bleeding in patients with hepatitis C-related cirrhosis [43]. The cohort was divided into two groups, cirrhotic patients with (n = 20) and without variceal bleeding (n = 20). Multiple parameters predicted bleeding: EXTEM CFT was significantly prolonged compared to the non-bleeding group (median = 392 seconds (IQR = 200.5-796) vs. median = 195 seconds (IQR = 123-309), respectively; p = 0.008); likewise, INTEM CFT was also significantly prolonged in the bleeding group (median = 251 seconds (IQR = 170-402) vs. median = 156 seconds (IQR = 100.5-207), respectively; p = 0.009). The EXTEM alpha angle was also prolonged compared to non-bleeders (mean = 52.7 degrees ± 17.4 vs. 65.5 degrees ± 9.9; p = 0.007). Furthermore, EXTEM A10 (mean = 25.1 ± 11.5 mm vs. 39.1 ± 12.7 mm; p = 0.001) and INTEM A10 (31.7 mm ± 11.9 vs. 43.1 mm ± 15.3; p = 0.012) significantly predicted variceal bleeding, as did EXTEM MCF (30 mm (IQR = 225-43) vs. 45 mm (IQR = 40-58); p = 0.002) [43]. Finally, INTEM MCF was significantly prolonged in bleeders compared to non-bleeders (median = 37.5 mm (IQR = 31-46) vs. median = 47 mm (IQR = 40-59); p = 0.014, respectively). Of note, the INR also distinguished patients at risk of variceal bleeding (p = 0.05), albeit to a lesser degree than the ROTEM parameters described above [43].

While various ROTEM parameters have shown the potential to predict bleeding in patients with liver disease, study limitations have prevented the construction of a unifying predictive algorithm. Shortcomings include the application of ROTEM across diverse clinical settings and the description of relatively small patient cohorts with very small numbers of bleeders in each study. These issues are compounded by the variation in threshold values used for individual VET parameters, which have, in turn, made difficult the ability to determine strengths of association to clinical outcomes. Whether the trends reported by studies conducted to date are held across the spectrum of severity and chronicity of liver disease has not been established (Table 2).

**Table 2:** Studies evaluating viscoelastic testing as a predictor of bleeding in liver disease. ACLF = acute-on-chronic liver failure; CT = clotting time; FFP = fresh frozen plasma; MCF = maximal clot firmness; RBC = red blood cell; TXA = tranexamic acid; SCT = standard coagulation tests

Study	Design	Study population	Method	Study groups	FFP or prothrombin complex concentrate (PCC) transfusion trigger (dose)	Cryoprecipitate transfusion trigger (dose)	Platelets transfusion trigger (dose)	RBC transfusion trigger (dose)	TXA transfusion trigger (dose)	Cryoprecipitate or fibrinogen concentrate transfusion trigger (dose)
Seeble et. al. (2020) [41]	Prospective	77 cirrhotic patients undergoing low bleeding risk procedures	Statistical analysis of blood results correlated to clinical outcomes	ACLF vs. non-ACLF, subgroup of ACLF bleeding vs. ACLF non-bleeding	Data on transfusion not recorded in the study					
Dotsch et. al. (2017) [42]	Retrospective, single-centre, observational study	243 liver transplantation cases	ROTEM and SCT performed on postoperative admission to intensive care unit and ROTEM-guided transfusion strategy followed	Postoperative bleeding vs postoperative non-bleeding	CT INTEM >240 seconds with clinically significant bleeding (10 mL/kg) 25 U kg^−1 ^was infused during 20 minutes if the CT_EXTEM _was >80 seconds, and 40 U kg^−1 ^if the CT_EXTEM _was >100 seconds		MCF FIBTEM >9 mm and MCF EXTEM <40 mm (1 apheresis or pooled unit)	Haemoglobin <7 g/dL	ROTEM-detected hyperfibrinolysis (25 mg/kg bolus)	MCF EXTEM reduced and MCF FIBTEM <9 mm (2 g fibrinogen concentrate) or <6 mm (4 g)
El-Makarem et. al. (2019) [43]	Retrospective	40 hepatitis C virus-related cirrhotic patients	Statistical analysis of blood results correlated to clinical outcomes	With vs. without variceal bleeding	No transfusions noted					

Currently, EASL does not recommend the use of VET to predict bleeding risk [26]. Other international haemostasis and gastroenterology societies have not made recommendations on the use of VET in this setting [30,44,45].

Viscoelastic goal-directed algorithms for the prophylaxis and treatment of bleeding in cirrhosis

Liver Transplantation

VET has been extensively explored as a tool to guide transfusion for patients with liver disease undergoing liver transplantation (Table 3). A trial conducted by Wang et al. randomised 28 liver transplant patients into a TEG analysis group or an SCT group [46]. Blood samples were obtained eight times throughout the intraoperative period, and transfusion was triggered by set values for each parameter. In comparison to the SCT group, ROTEM-guided transfusion resulted in significantly less FFP use (mean = 12.8 ± 7.0 units vs. 21.5 ± 12.7 units, p < 0.05) [46]. All other blood products showed an insignificant reduction in transfusion, cryoprecipitate (13.0 ± 10.3 units vs. 15.6 ± 9.5 units), platelet transfusion (27.3 ± 13.9 units vs 30.1 ± 18.5 units), and packed RBCs (14.2 ± 7.1 units vs. 16.7 ± 12.8 units) [46]. An insignificant reduction in intraoperative blood loss (mean 4,775.7 ± 4,264.7 mL vs. 6,348.0 ± 3,704.1 mL) and total fluid administration (9,198.0 mL vs. 100,053.8 mL) was also recorded [46].

**Table 3:** Studies investigating viscoelastic testing goal-directed algorithms in liver transplantation. CT = clotting time; FFP = fresh frozen plasma; Hb = haemoglobin; INR = international normalised ratio; MA = maximum amplitude; MCF = maximal clot firmness; RBC = red blood cell; RCT = randomised control trial; R time = reaction time; TEG = thromboelastograpthy; TXA = tranexamic acid; SCT = standard coagulation tests

Study	Design	Study population	Method	Study groups	FFP transfusion trigger (dose)	Cryoprecipitate transfusion trigger (dose)	Platelets transfusion trigger (dose)	RBC transfusion trigger (dose)	TXA transfusion trigger (dose)	Fibrinogen transfusion trigger (dose)
Wang et al. (2010) [46]	RCT	28 patients undergoing liver transplantation	8 intraoperative blood samples with transfusion trigger values of TEG and SCT parameters	Point-of-care TEG	R time >10 minutes	Alpha angle <45 degrees (5 units)	MA <55mm (1 apheresis unit)	Hb <8 g/dL		
				SCTs	INR >1.5	Fibrinogen concentration <1 g/L	Maintain platelet count of 50 × 10^9^/L	Hb <8 g/dL		
Leon-Justel et al. (2015) [47]	Prospective	200 consecutive liver transplant patients	4 intraoperative samples with pre-determined algorithms guiding transfusion amounts	100 ROTEM-guided cases	EXTEM A10 <35 mm MCF <35 mm		FIBTEM MCF >8 mm or FIBTEM MCF >6 mm and EXTEM MCF <32 mm	Hb <7 g/dL	%Lysis >15 (1 g IV bolus)	FIBTEM MCF <6 mm
				100 SCT-guided cases	INR >1.6		Platelet count <70 × 10^9^/L	Hb <7 g/dL		Fibrinogen <1 g/L
Zamper et al. (2018) [48]	Before-after	237 liver transplant patients with chronic disease aetiology	A preoperative, intraoperative, and postoperative sample with transfusions enacted for each	54 intervention ROTEM patients with transfusion triggered by parameters becoming abnormal	INTEM CT >240 seconds and if HEPTEM CT > INTEM CT (2–4 units)	EXTEM A5 <25 mm and FIBTEM A10 <10 mm		Hb <7 g/dL	EXTEM CLI30 >50% after reperfusion (15 mg/kg)	EXTEM A5 <25 mm and FIBTEM A10 <10 mm
				183 control patients	Not described					
Kandeel et al. (2018) [49]	Before-after	216 liver transplant cases classified at high risk of massive intraoperative bleeding	ROTEM-guided algorithm with a sample taken preoperatively and transfused accordingly preoperatively	121 ROTEM-guided algorithm cases	EXTEM CT >80 seconds and HEPTEM CT >280seconds (30 mL/kg)		EXTEM A10 <45 mm and FIBTEM A10 >15 mm	Hb <8 g/dL or 10 g/dL if ischaemic heart disease	EXTEM ML >15% (2 g IV bolus)	EXTEM A10 <45 mm and FIBTEM A10 <15 mm (2–6 g)
				95 Pre-ROTEM algorithm	INR >2.0 (15 mL/kg)		1:1 with FFP in massive bleeding	1:1 with FFP in massive bleeding		

A study conducted by Leon-Justel et al. [47] showed that a VET-based transfusion strategy not only reduced allogenic blood product use but also reduced the incidence of postoperative complications that included reoperation for bleeding, acute renal failure, and haemodynamic instability [47]. Here, investigators compared ROTEM-guided transfusion to an SCT group to explore the impact of these approaches on transfusion requirements per patient intraoperatively. In total, 100 consecutive patients were recruited into each group. The median units of RBCs transfused were reduced from 5 to 3 (p < 0.001), and blood component usage including FFP and platelets were reduced from 2 to 0 (p < 0.001) and 1 to 0 (p < 0.001), respectively [47]. The use of fibrinogen concentrate was higher in the ROTEM group (1.13 ± 1.44 g vs. 0.48 ± 1.28 g, p = 0.001). Tranexamic acid was required in four patients in the POC group and in one patient in the SCT group (p = 0.369) [47]. Importantly, transfusion was completely avoided in 5% vs. 24% (p < 0.001), and the incidence of massive transfusion (defined as the transfusion of more than 10 RBC units) was reduced from 13% vs. 2% (p = 0.005) [47].

A more recent before-after cohort study conducted by Zamper et al. [48] among 237 liver transplant patients compared a retrospective control SCT-based algorithm (n = 183) and an interventional prospective ROTEM-guided transfusion algorithm (n = 54). Other than prophylactic tranexamic acid that was infused preoperatively, all blood products were delivered intraoperatively in response to real-time ROTEM traces drawn preoperatively and throughout the intraoperative period. The proportion of transfusion of any blood component (RBCs, FFP, cryoprecipitate, and platelets) reduced from 56.3% to 35.2% in the intervention group (p = 0.006) [48]. RBC transfusion reduced from 52.5% to 30.2% (p = 0.004) (mean = 1.7 ± 2.7 units vs. 0.7 ± 1.3 units, p = 0.007). FFP was transfused in 27.3% of control patients and only 5.7% of intervention patients (p < 0.001) (mean = 2.1 ± 4.2 units vs. 0.2 ± 0.8 units, respectively, p = 0.001) [48]. Cryoprecipitate was transfused at a very similar rate (0.4 ± 1.8 units in control vs 0.4 ± 2.1 units in intervention, p = 0.876), as were platelets (0.2 ± 0.4 apheresis units in both groups, p = 0.871) [48]. Components such as fibrinogen concentrate, tranexamic acid, and prothrombin complex concentrate were transfused at much higher rates in the intervention group, with 35.2% receiving one or more of these products compared to 0% of the control group (p < 0.001) [48]. There was a slight but insignificant reduction in procedural complications in the ROTEM group, with 54.1% reduced to 47.2% (p = 0.373) [48]. The main complication was upper digestive tract haemorrhage, which occurred in the control group in 31.0% of cases compared to 18.9% of intervention cases (p = 0.084). Arterial thrombosis occurred in 3.5% of the control cohort compared to 1.9% of the intervention group; however, this was not significant (p = 0.557) [48].

Another retrospective study by Kandeel et al. investigated the effect of a prophylactic ROTEM-guided on blood product transfusion practice in living donor liver transplantation recipients [49]. Investigators screened 380 cases and identified 216 patients who had one or more preoperative predictors of massive intraoperative bleeding (bleeding >70 mL blood/kg; INR = 2, platelet count <50 × 10^9^, haemoglobin <8 g/dL, Model for End-Stage Liver Disease score >30, serum albumin <2.5 g/dL). In total, 95 patients were included in the pre-ROTEM group and 121 were included in the ROTEM-guided group. Transfusion of RBCs, FFP, and massive transfusion significantly reduced in the ROTEM group compared to the pre-ROTEM group (median = 8 units vs. 4.5 units, p < 0.01; 12.5 units vs. 5.6 units, p < 0.001; and 29% vs. 20%, p < 0.05, respectively). No significant difference in one-year survival was observed between the two groups (17% vs. 21% in the ROTEM group) [49].

Taken together, studies to date have shown a marked impact of VET-guided protocols in reducing blood product requirements and bleeding in this setting, supporting its recommendation in societal guidelines [45,50].

Invasive Procedures Other Than Liver Transplantation

Other clinical trials have focused on prophylactic transfusion strategies during a variety of procedures performed in patients with chronic liver disease (Table 4). De Pietri et al. studied 60 patients with chronic liver disease and divided the cohort into a TEG-guided (n = 30) and standard of care (SOC) (n = 30) to receive prophylactic transfusion strategies for high (>3% risk of procedure-related bleeding) and low (<3% risk of procedure-related bleeding) bleeding risk procedures [51]. The main contingencies of the low-risk procedures were paracentesis and thoracentesis, while high-risk procedures included variceal banding, hepatic resection, radiofrequency ablations, and miscellaneous abdominal surgeries among others. FFP and platelets were the only blood product transfused in this study. The overall use of blood products was significantly lower in the TEG group (16.7% vs. 100% in the SOC group, p < 0.0001) [51]. The total amount of FFP transfused across each group was significantly lower in the TEG group with 4,000 mL, while 11,050 mL was transfused to patients in the SOC group (p = 0.002) [51]. Platelet usage was also significantly reduced; the TEG group received a total of six pooled units compared to 78 units in the SOC group (p = 0.001) [51]. There was one case each of a transfusion-related side effect and procedure-related bleeding in the SOC group (both 3.3% incidence), but it was non-significant (p = 0.313) [51]. Ninety-day mortality was 26.6% and 23.3% in the TEG and SOC groups, respectively (p = 0.880) [51].

**Table 4:** Studies investigating viscoelastic testing algorithms in the setting of invasive procedures other than liver transplantation. CT = clotting time; FFP = fresh frozen plasma; Hb = haemoglobin; INR = international normalised ratio; MA = maximum amplitude; MCF = maximal clot firmness; RBC = red blood cell; RCT = randomised control trial; R time = reaction time; TEG = thromboelastograpthy; SCT = standard coagulation tests; SOC = standard of care

Study	Design	Study population	Method	Study groups	FFP transfusion trigger (dose)	Cryoprecipitate transfusion trigger (dose)	Platelets transfusion trigger (dose)
De Pietri et al. (2016) [51]	RCT	60 chronic liver disease patients getting low and high-risk procedures	TEG-guided and control SCT-guided algorithms compared being applied preoperatively	TEG-guided	R time >40 minutes (10 mL/kg)		MA <30 mm (1 apheresis unit)
				Standard-of-care (SOC) group	INR >1.8 (10 mL/kg)		Platelet count <50 × 10^9^/L (1 apheresis unit)
Vuyyuru et al. (2020) [52]	RCT	58 cirrhosis patients with coagulopathy undergoing high-risk invasive procedures	TEG-guided and control SCT-guided algorithms compared being applied preoperatively	TEG-guided	R time >14 minutes (10 mL/kg)		MA <32 mm (3 units)
				SOC group	INR >1.8 (5 mL/kg)		Platelet count <50 × 10^9^/L (3 units)
Rocha et al. (2017) [53]	RCT	57 central venous catheterisation chronic liver disease patients	ROTEM-guided and control SCT-guided algorithms compared being applied preoperatively	ROTEM-guided	EXTEM CT >80 seconds (10 mL/kg)	A10 EXTEM <40 mm and A10 FIBTEM <10 mm (1 unit/10 kg until 10 units)	A10 EXTEM <40 mm and A10 FIBTEM ³ 10 mm (1 apheresis unit)
				SCT-based algorithm	INR >1.5 or aPTT >50 seconds (10 mL/kg)	Fibrinogen <150 mg/dL (1 unit/10 kg until 10 units)	Platelet count <50 × 10^9^/L (1 apheresis unit)
				Restrictive protocol (based on SCT but with wider transfusion triggers)	INR >5.0 (10 mL/kg)		Platelet count <25 × 10^9^/L (1 apheresis unit)

Vuyyuru et al. conducted an unblinded randomised controlled trial (RCT) to assess a TEG-guided transfusion algorithm compared to an SCT-guided transfusion in 58 patients with cirrhosis undergoing high bleeding risk (> 3% risk of procedure-related bleeding) invasive procedures such as percutaneous liver biopsy, transarterial chemoembolization, and pigtail drainage [52]. Low-risk (<3% risk of procedure-related bleeding) procedures such as transjugular liver biopsy, abdominal paracentesis, and central line insertion were excluded. SCTs as well as TEG analysis were performed before the procedure and transfusions were based on pre-defined parameters (Table 4). Overall, supplementation with platelet transfusion, FFP, or both was required by 31% of patients in the TEG group compared to 100% in the SCT group (p = 0.001) [52]. Overall, 6.9% of participants in the TEG group received platelet transfusions compared to 72.4% in the SCT group (p < 0.001) [52]. There was no difference in FFP transfusion requirement between patients receiving TEG and SCT-guided strategies (20.6% vs. 24.1%, respectively, p = 0.753). No patients developed procedure-related bleeding complications within five days post-procedure [52].

A more restrictive SCT protocol was studied in patients undergoing low-risk therapeutic procedures. This double-blinded RCT by Rocha et al. studied 57 critically ill cirrhotic patients undergoing central venous catheterisation (CVC) [53]. The cohort was divided into three groups of 19 patients each, namely, ROTEM-guided, SCT-guided, and restrictive SCT-guided groups (Table 4). The SCT group used prophylactic trigger values, INR >1.5 or aPTT >50 seconds, for FFP transfusion, platelets were transfused when platelet count was <50 × 10^9^/L, and cryoprecipitate was transfused when fibrinogen was <150 mg/dL. The restrictive SCT group received FFP if the INR was >5 and platelets if the platelet count was <25 × 10^9^/L. Of patients in the SCT group, 73.7% received prophylactic blood products compared to 68.4% in the ROTEM group and 15.8% in the restrictive group (restrictive vs. SCT, p = 0.002; restrictive vs, ROTEM, p = 0.006) [53]. While no patient in any group had major bleeding, 21% of patients in the restrictive group and 10.5% in the SCT group experienced minor bleeding, while no patients in the ROTEM group experienced bleeding [53].

Gastrointestinal Bleeding 

A recent RCT by Rout et al. evaluated TEG-guided blood product transfusion in patients with cirrhosis and variceal bleeding, and designated the cohort to receive either TEG-guided or SCT-guided transfusion replacement [54]. The algorithm for both groups only included replacement with FFP, platelets, and RBCs. In the TEG group, 13.3% received blood products compared to 100% in the SCT-guided transfusion group (p < 0.001). The total FFP volume transfused reduced from 4,605.0 mL in the conventional group to 1,345.0 mL. FFP was transfused in 13.3% of patients in the TEG-guided group and 46.7% in the conventional group (p = 0.010) [54]. There was also a significant reduction in the proportion of patients receiving platelets, with only 10% in the TEG group compared to 70% in the conventional group (p < 0.001) [54]. No significant difference was observed in RBC transfusion between the groups [54]. While the groups had similar rates of rebleeding at day five post-procedure, after 42 days there was significantly less rebleeding in the TEG group (10% vs. 36.7%, p = 0.012) [54]. Mortality at six weeks was similar between groups (13.3% in the TEG group vs. 26.7% in the conventional group, p = 0.176). No significant difference was observed in the rate of transfusion reactions [54].

Regarding non-variceal gastrointestinal bleeding, an RCT by Kumar et al. assessed 96 patients with cirrhosis and coagulopathy (defined as INR >1.8 and platelet count <50 × 10^9^/L) and ongoing bleeding from a non-variceal source. The cohort was allocated to a TEG-guided (n = 49) or an SOC-guided (n = 47) approach [55]. Transfusion in the SOC group was triggered by INR and platelet parameters (Table 5). Both the amount of blood product usage and clinical outcomes (transfusion-related reactions and five-day treatment failure (failure to control bleeding)) were analysed. The mean volume of FFP transfused per patient was 440 mL in the TEG group compared to 880 mL in the SOC group (p < 0.001) [55]. Platelet transfusion was also reduced in the TEG group (median = 1 apheresis unit vs. 2 apheresis units in the SOC group, p < 0.001). Cryoprecipitate transfusion was significantly lower in the TEG group (median = 4 units vs. 16 units, p < 0.001). Five-day treatment failure showed a slight but insignificant reduction for the TEG group (22.4 vs. 29.8%, p = 0.488). There was also a significant reduction in the incidence of transfusion-related reactions in the TEG group (30.6% vs. 74.5%, p < 0.001). The incidence of transfusion-related acute lung injury and acute respiratory distress syndrome was significantly reduced in the TEG group (12.2% vs. 48.9%, p < 0.001) [55].

**Table 5:** Summary of studies evaluating the efficacy of viscoelastic testing-guided algorithms in the context of gastrointestinal bleeding. FFP = fresh frozen plasma; Hb = haemoglobin; INR = international normalised ratio; MA = maximum amplitude; RBC = red blood cell; RCT = randomised control trial; R time = reaction time; TEG = thromboelastograpthy; SCT = standard coagulation tests; SOC = standard of care

Study	Design	Study population	Method	Study groups	FFP transfusion trigger (dose)	Cryoprecipitate transfusion trigger (dose)	Platelet transfusion trigger (dose)	RBC transfusion trigger (dose)
Rout et al. (2020) [54]	RCT	60 cirrhotic patients with severe coagulopathy and acute variceal bleeding	Preoperative sample taken and used to guide the TEG and SOC transfusion	TEG-guided transfusion	R time >15 minutes (5 mL/kg)		MA <30 mm (3 units)	Hb <7 g/dL
				SOC-based transfusion	INR >1.8 (5 mL/kg)		Platelet count <50 × 10^9^/L (3 units)	Hb <7 g/dL
Kumar et al. (2020) [55]	RCT	96 patients with ongoing non-variceal upper gastrointestinal bleeding	Preoperative sample taken and used to guide the TEG and SOC transfusion	TEG-guided transfusion	R time >10 minutes (10 mL/kg)	Alpha angle <45 degrees (5 pooled units)	MA <55 mm (1 apheresis unit)	
				SOC-based transfusion	INR >1.8 (10 mL/kg)	Fibrinogen concentration <80 mg/dL (5 pooled units)	Platelet count < 50 × 10^9^/L (1 apheresis unit)	

In summary, there have been favourable outcomes reported following the uptake of TEG in cohorts of variceal and non-variceal gastrointestinal bleeding. Studies have documented a global reduction in blood product usage, and various outcomes such as rebleeding and transfusion reactions were reported to decrease with a TEG-guided algorithm. While more data are required in this setting, these results indicate a potential benefit of using VET-guided algorithms in this context. This sentiment is echoed by the EASL clinical practice guidelines supporting the use of VET to reduce the use of blood products in patients with cirrhosis and active upper gastrointestinal bleeding [26].

Non-gastroenterological Surgeries in Patients With Liver Disease

To our knowledge, cardiac surgery, spinal surgery, trauma, and other surgeries in patients with liver disease have not been specifically investigated. Given the intricacies of haemostatic balance in patients with liver disease and the potential necessity to undergo surgeries for comorbid conditions, evidence-based recommendations for managing their bleeding and thrombotic risk may assist clinicians in providing better care and remains an area for future research.

Overall, there appears to be a beneficial role of a VET-guided approach to transfusion among patients with cirrhosis across various settings studied to date. A recent meta-analysis attempted to quantitate this effect by analysing all clinical trials comparing SCT versus VET for the assessment and reversal of coagulopathy among patients with cirrhosis [56]. Seventeen studies were included in the final analysis. Four of the included studies described a pre-procedural setting, four studies included patients with acute bleeding, and nine studies were in the liver transplantation setting. The analysis confirmed that VET-guided transfusion protocols reduced the number of patients transfused, as well as the amount of transfused packed RBCs, platelets, and FFP. There was a trend towards increased cryoprecipitate transfusion. Moreover, bleeding events were significantly reduced in the VET-guided group and there was a trend towards less transfusion-related reactions and cost.

Algorithms

As detailed above (Tables 2-5), there is currently no standardised VET-guided algorithmic approach to guide clinicians, and studies have employed differing algorithms with wide variations in normal ranges and parameters for transfusion. Studies exploring VET to guide the treatment of bleeding show the most marked differences in algorithmic parameters [46-48], while those that explored VET in the prophylactic setting have more in common. In the latter group, the transfusion trigger for R time tends to be between 10 and 15 minutes, an EXTEM CT cut-off of 80 seconds is common for triggering 5-10 mL/kg of FFP, 1 apheresis unit of platelets tends to be transfused using triggers of either EXTEM A10 <40-45 mm and FIBTEM A10 >10 mm or FIBTEM MA <30-32 mm [49,51-55]. However, there is no common thread or set parameter value that triggers transfusion, which emphasises the lack of cohesion of algorithms and the need for a coordinated response to define the population most at risk of bleeding and to develop an algorithm that utilises standardised transfusion parameters and doses.

Viscoelastometry testing in predicting thrombotic events

The capacity of VET to predict thrombotic events in patients with liver disease has been explored in several trials. A prospective observational study by Hinckler et al. analysed the preoperative samples of 313 non-cardiac surgical patients. Routine anti-coagulation measures were taken by the treating physicians, who were not given access to the ROTEM results. ROTEM parameters were found to have good predictive power for predicting thromboembolic complications [57]. Standard coagulation tests such as PT, INR, or platelet count failed to predict thrombotic complications. Investigators noted that several ROTEM parameters were predictive, including EXTEM: CFT (mean ± SD of 58.5 ± 16.6 seconds when thrombosis occurred vs. 87.1 ± 61.7 seconds when it did not, p < 0.001), alpha angle (78.4 ± 3.1 degrees with thrombosis vs. 74.3 ± 6.9 degrees without, p = 0.002), A10 (64.0 ± 5.4 mm with vs. 58.2 ± 8.7 mm without, p = 0.008), and MCF (70.4 ± 5.2 mm with vs 65.0 ± 7.5 mm without, p = 0.009) [57]. The same parameters were significant using INTEM: CFT (51.0 ± 11.4 seconds with vs. 72.2 ± 57.3 seconds without, p < 0.001), alpha angle (79.4 ± 2.4 degrees with thrombosis vs. 76.5 ± 5.5 degrees without, p = 0.006), A10 (63.0 ± 5.8 mm with vs. 56.7 ± 7.8 mm without, p = 0.012), and MCF (68.6 ± 6.0 mm with vs. 62.8 ± 7.1 mm without, p = 0.020) [57]. FIBTEM also showed significance: A10 (23.7 ± 11.2 mm with vs. 16.5 ± 6.8 mm without thrombosis, p = 0.01) and MCF (24.8 ± 11.2 mm with vs. 17.8 ± 7.6 mm, p = 0.015) [57]. Overall, 6% of cases included patients undergoing hepatic surgery. In this group, 1 in 20 patients developed a thrombotic complication, although there was no confirmation that underlying liver disease was the indication for surgery [57]. A more recent meta-analysis by Harahsheh et al. included 41 relevant studies that supported ROTEM and TEG in predicting thromboembolic events in any setting, including hospitalised patients not undergoing a procedure, trauma patients, and patients undergoing orthopaedic, neurological, cardiothoracic, oncological, and liver transplantation surgery [58]. VET was credited with a better ability to predict arterial thromboembolic events (sROC = 0.75, 95% CI = 0.65-0.84) than deep vein thrombosis or pulmonary embolism (sROC = 0.68, 95% CI = 0.61-0.75) [58]. VET had a moderate ability to predict portal vein thrombosis in cirrhotic patients (sROC = 0.75, 95% CI = 0.61-0.88, sensitivity = 21%, specificity = 78%, pooled diagnostic odds ratio = 4.4, 95% CI = 1.7-11.1) [58]. The MCF parameter across the EXTEM, INTEM, and FIBTEM tests was commonly used to detect hypercoagulability, and this parameter was moderately effective in differentiating patients who had thromboembolic complications and those without, leading to the overall conclusion that ROTEM had clinical utility in assessing the risk of thrombotic events [58].

These studies support the potential role of VET in predicting thrombotic complications across a variety of surgical settings; however, the role of VET for this purpose in patients with liver disease undergoing invasive procedures is yet to be determined. This lack of data, coupled with concern that VET may underestimate hypercoagulability in patients with cirrhosis due to its insensitivity to detect raised levels of VWF and the anti-coagulant action of the protein C system, were the main factors cited by EASL supporting their negative recommendation of VET to identify patients with cirrhosis at risk of venous thromboembolism [26].

Early hepatic artery thrombosis (e-HAT) following liver transplantation negatively impacts graft function and patient survival. A retrospective study by Eldeen et al. evaluated the utility of preoperative TEG in predicting postoperative e-HAT in a large cohort of patients undergoing liver transplantation [59]. Overall, 9.5% of the total cohort was diagnosed with HAT, 2.8% experiencing e-HAT and 6.7% late HAT [59]. The MA on the preoperative TEG was significantly higher in patients diagnosed with e-HAT compared to those who were not (71.2 mm vs. 57.9 mm, p < 0.0001) [59]. A TEG MA cut-off value of 65 mm had a sensitivity of 70% for predicting; 7% of patients with an MA equal to or over 65 mm developed e-HAT (p < 0.001), while 1.2% with an MA below 65 mm experienced e-HAT [59]. The median MA in the e-HAT group was 71.2 mm (IQR = 62.5-77.6) compared to 63.3 mm (IQR = 53.4-69.2) for patients without HAT [59].

A retrospective analysis by Krzanicki et al. also explored TEG’s ability to predict HAT by sampling blood at various intervals throughout the operation [60]. Overall, 5% of cases experienced HAT, 50% of these cases having high G traces (a TEG parameter for clot elasticity, net clot strength) (p = 0.25), and 66% of the cases having shortened R times (reaction times) (p = 0.76) [60]. Furthermore, 66% of patients with HAT were found to have hypercoagulable traces by TEG. Variations were found between liver disease aetiology. An increased incidence of high G values was found in patients with underlying primary sclerosing cholangitis and primary biliary cirrhosis (85% and 43% were above the G value reference ranges, respectively). Patients with alcoholic or viral liver disease had short or hypercoagulable R times in 65-100% of the cases, but only 10-12% had increased G values. Additionally, only two of 26 patients with hepatocellular carcinoma had high G values [60].

Overall, early data suggest that TEG parameters have a positive correlation with the development of HAT in patients undergoing liver transplantation surgery, though the degree of correlation may vary with the underlying aetiology of liver disease (Table 6).

**Table 6:** Studies of viscoelastic testing in predicting thrombotic events. HAT = hepatic artery thrombosis; TEG = thromboelastography; VET = viscoelastic testing

Study	Design	Study population	Study groups	Method	Findings in VET-guided group compared to control
Hincker et al. (2014) [57]	Prospective observational	Non-cardiac surgical patients – 6% of the population is relevant having undergone hepatic surgery	Not separated at collection, statistical analysis carried out on patients with vs. without thromboembolic complications once followed up	Preoperative samples taken and results regarding hypercoagulability correlated to post-procedural thromboembolic complications	1 in 20 patients who underwent hepatic surgery developed a thrombotic complication. Indication for surgery not known
Harahsheh and Ho (2017) [58]	Meta-analysis	ROTEM in any setting	41 included studies	-	88% of studies used maximum clot strength to identify hypercoagulability. It was moderately effective at predicting thromboembolic events
Eldeen et al. (2016) [59]	Retrospective	828 adult liver transplantation patients	Patients who developed early HAT postoperatively vs. those who did not	Preoperative TEG sample taken, and the outcome of postoperative early HAT correlated to TEG results	Maximum amplitude (MA) was significantly higher in early HAT patients compared to unaffected group (71.2 mm vs. 57.9 mm, p < 0.0001). Using a cut-off value of 65 mm for MA predicted early HAT with 70% sensitivity
Krzanickiet al. (2013) [60]	Retrospective	124 liver transplant recipients	Patients who developed HAT postoperatively vs. those who did not	Multiple samples taken throughout the operative period and correlated to postoperative thrombotic outcomes. A TEG-guided transfusion algorithm was used throughout	5% experience HAT – 50% of these cases had high G traces and 66% had shortened R times. Higher incidence of high G values in primary sclerosing cholangitis and primary biliary cirrhosis

## Conclusions

VET provides clinicians with a means to assess global haemostasis in patients with liver disease. Interest in VET platforms has grown tremendously over the last few decades with the unrefuted demonstration of the limitations of standard coagulation tests to predict bleeding risk in these patients. To date, VET has shown utility in reducing blood product usage in patients undergoing liver transplantation. Its use, however, to predict and prevent bleeding in patients undergoing invasive procedures (other than liver transplantation) has shown less consistent benefits. Overall, standardised terms to define hypocoagulability and/or hypercoagulability and therefore overall bleeding/clotting risk have not been established and so thresholds to determine transfusion of blood products have varied among studies making the significance and applicability of findings hard to apply. In addition, the blood product used and the dose of blood product infused when a threshold is crossed lack clear definition and has tended to vary among study populations/geographical areas. Currently, the peak professional bodies have no clear recommendations on the use of VET. To move forward, the field needs to address these limitations to optimally harness the potential of these platforms to provide better predictive, treatment, and prognostic information in patients with liver disease.
